# Whole-Exome Sequencing in Patients with Tooth Agenesis and Neurodevelopmental Disorders: A Descriptive Case Series

**DOI:** 10.3390/ijms27209357

**Published:** 2026-10-21

**Authors:** Francesco Calì, Antonino Musumeci, Mirella Vinci, Simona L’Episcopo, Massimiliano Bartolone, Angelo Gloria, Raffaele Ferri, Simone Treccarichi, Antonio Fallea

**Affiliations:** 1 Oasi Research Institute-IRCCS, 94018 Troina, Italy; cali@oasi.en.it (F.C.); amusumeci@oasi.en.it (A.M.); mvinci@oasi.en.it (M.V.); slepiscopo@oasi.en.it (S.L.); mbartolone@oasi.en.it (M.B.); agloria@oasi.en.it (A.G.); rferri@oasi.en.it (R.F.); 2 Department Medicine and Surgery, Kore University of Enna, 94100 Enna, Italy

**Keywords:** tooth agenesis, dental agenesis, neurodevelopmental disorders, next generation sequencing

## Abstract

Tooth agenesis (TA) is a common developmental abnormality that may occur as an isolated trait or as part of a multisystem disorder. Its genetic architecture remains incompletely characterized, particularly in individuals with neurodevelopmental disorders (NDDs). This descriptive case series aimed to investigate potentially relevant genetic variants in a clinically selected cohort of patients with TA using whole-exome sequencing (WES). Ten patients with TA and neurological or neurodevelopmental conditions were recruited at the IRCCS Oasi Research Institute of Troina between January 2024 and July 2026. The cohort included eight males and two females (age range: 5–50 years). The associated clinical spectrum included autism spectrum disorder (ASD), intellectual disability (ID), language impairment, behavioral abnormalities, and Down syndrome (DS). WES was performed in the examined cohort and variants were prioritized according to population frequency, predicted functional impact, inheritance pattern, and phenotypic concordance. The cohort comprised nine patients with hypodontia and one with oligodontia. Thirty variants involving 28 genes were identified. A homozygous *GLI3* p.Gly727Arg variant, inherited from two heterozygous parents, was detected in an eight-year-old boy with ASD, global developmental delay, and hypodontia. Pathogenic or likely pathogenic loss-of-function variants in *BCL11B* were identified in two girls with ID and hypodontia. A likely pathogenic *NIPBL* splice-site variant was identified in a 15-year-old boy with hypodontia. His similarly affected father carried a novel FGFR1 p.Asn185His variant that was absent in the son, excluding it as the sole explanation for the familial dental phenotype. A 50-year-old man with DS and oligodontia carried the recurrent pathogenic BBS1 p.Met390Arg allele in the heterozygous state, together with variants of uncertain significance in *PTH1R* and *STAT3*. However, none of these variants independently explained his dental phenotype. The remaining variants were primarily relevant to the associated neurological phenotypes or represented secondary findings. This descriptive case series illustrates the clinical and genetic heterogeneity of TA in individuals with NDDs. Although the findings do not establish causal relationships with TA, they highlight the importance of distinguishing the general clinical significance of a variant from its specific relevance to dental development. Replication in larger cohorts and functional validation will be required to clarify the contribution of the identified candidate variants.

## 1. Introduction

Tooth agenesis (TA) is a developmental anomaly characterized by the congenital absence of one or more teeth because of failure of tooth-germ formation [1,2]. It represents one of the most common abnormalities of human dentition and may affect both primary and permanent teeth, although the latter are more frequently involved. Beyond its aesthetic consequences, TA may impair mastication, speech, occlusion, and craniofacial development, frequently requiring long-term multidisciplinary management involving pediatric dentists, orthodontists, prosthodontists, and oral surgeons [3,4]. TA can be classified according to both the number of missing teeth and the presence of additional clinical manifestations. Hypodontia commonly refers to the congenital absence of one to five permanent teeth, whereas oligodontia is defined by the absence of six or more permanent teeth; third molars are generally excluded from this classification [5,6]. Anodontia denotes the complete absence of the dentition and represents the rarest and most severe form. Dental assessment and management in patients presenting with NDDs may also be complicated by odontophobia, which can reduce patient cooperation, delay access to dental care, and interfere with diagnostic and therapeutic procedures [7]. From a clinical perspective, TA may occur as an isolated, non-syndromic trait or as part of a syndromic disorder involving additional ectodermal, craniofacial, skeletal, or systemic abnormalities [8]. However, the distinction between syndromic and non-syndromic forms is not always clear, because different variants in the same gene may result in variable expressivity and a broad phenotypic spectrum [9]. The genetic architecture of TA is highly heterogeneous and includes autosomal dominant, autosomal recessive, and X-linked inheritance patterns [8,10]. Pathogenic variants have been identified in genes involved in key developmental pathways governing epithelial–mesenchymal interactions during odontogenesis, particularly WNT/β-catenin, TGF-β/BMP, EDA/EDAR/NF-κB, and SHH signaling. Among the genes most frequently implicated are *WNT10A*, *WNT10B*, *PAX9*, *MSX1*, *AXIN2*, *LRP6*, *EDA*, *EDAR*, and *EDARADD* [10,11,12,13,14]. Nevertheless, several genes initially associated with isolated TA have subsequently been linked to syndromic conditions, highlighting the clinical and molecular overlap between these categories. Incomplete penetrance, variable expressivity, and possible oligogenic contributions further complicate genotype–phenotype correlations [15,16]. The marked genetic heterogeneity of TA limits the effectiveness of sequential single-gene testing. Next-generation sequencing (NGS) approaches, including multigene panels, whole-exome sequencing (WES), and whole-genome sequencing (WGS), enable the simultaneous evaluation of multiple established and candidate genes [15,17]. In particular, trio-based WES facilitates the identification of de novo, recessive, and X-linked variants and allows segregation data to be incorporated directly into variant interpretation. These approaches may also reveal atypical or incompletely expressed syndromic conditions in patients initially presenting with apparently isolated dental abnormalities. Nevertheless, the identification of variants of uncertain significance (VUS) and secondary findings remains a major interpretative challenge and requires careful integration of genetic, segregation, clinical, and functional evidence.

In this descriptive case series, we applied trio-based WES to a cohort of patients with hypodontia or oligodontia and concomitant neurodevelopmental or neurodegenerative phenotypes. We aimed to characterize the genetic findings, evaluate their potential relevance to TA, and descriptively annotate the biological processes associated with the prioritized genes.

## 2. Results

### 2.1. Clinical and Dental Characteristics of the Study Cohort

**Figure 1:**
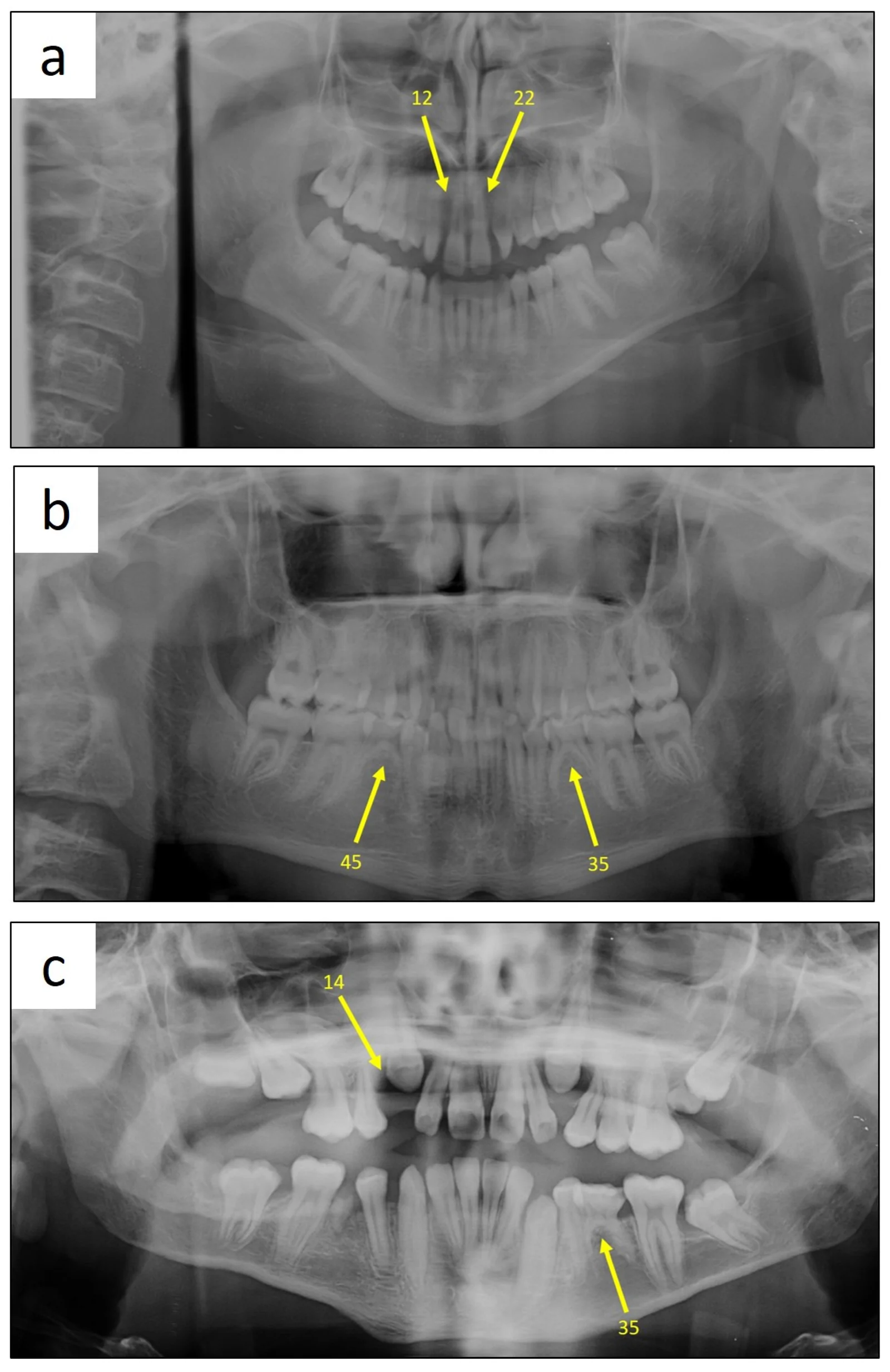
Representative panoramic radiographs of dental agenesis in three patients included in the study. (**a**) Patient P9, showing bilateral agenesis of the maxillary permanent lateral incisors (teeth 12 and 22). (**b**) Patient P6, showing agenesis of the mandibular permanent second premolars (teeth 35 and 45), with persistence of the deciduous tooth 85. (**c**) Patient P8, showing agenesis of the maxillary right first premolar (tooth 14) and mandibular left second premolar (tooth 35), with persistence of the corresponding deciduous tooth 75. Yellow arrows indicate the sites of the congenitally missing permanent teeth. Dental elements are reported according to the FDI World Dental Federation notation.

The study included ten patients with dental agenesis, comprising eight males and two females, with ages ranging from 5 to 50 years (mean 22 ± 17.04 years). Dental assessment identified hypodontia in nine patients and oligodontia in one patient. No cases of anodontia were identified. According to the Fédération Dentaire Internationale (FDI) notation, P1, P2, and P5 each showed agenesis of a single permanent tooth (12, 24, and 22, respectively). Two missing permanent teeth were observed in P3 (12 and 22), P6 (35 and 45), P7 (12 and 22), P8 (14 and 35), P9 (12 and 22), and P10 (12 and 22). P4 presented with oligodontia, involving the congenital absence of seven permanent teeth (14, 15, 22, 25, 34, 35, and 42). Overall, the maxillary lateral incisors, particularly teeth 12 and 22, were the most frequently absent elements in the cohort. All the phenotypic features regarding the cohort examined in this study are listed in Table S1. Representative panoramic radiographs illustrating the patterns of hypodontia observed in patients P9, P6, and P8 are shown in Figure 1.

### 2.2. Genetic Findings

WES identified 30 prioritized variants involving 28 genes across the 10 patients. Four variants were initially categorized as additional or secondary findings: *SLC22A5* c.113C>A p.Ser38Tyr, *GJB2* c.35del p.Gly12ValfsTer2, *PKP2* c.1881del p.Lys628ArgfsTer12, and *LPA* c.1173C>A p.Cys391Ter. These genes are associated with primary carnitine deficiency, hereditary hearing loss, arrhythmogenic cardiomyopathy, and lipoprotein(a)-related traits, respectively. However, the heterozygous *SLC22A5* and *GJB2* variants should primarily be considered carrier findings for autosomal recessive disorders, whereas the *PKP2* variant represents a potentially actionable cardiovascular finding. These variants were considered separately from the analysis of the dental phenotype. The broad spectrum of variants summarized in Table S2 highlights the marked genetic heterogeneity of this cohort and the complexity of distinguishing potentially odontogenesis-related variants from findings associated with the patients’ broader phenotypes.

Four splice-region variants were further evaluated using SpliceAI, Pangolin, and SAI-10k-calc. Concordant evidence of splice-site disruption was obtained for the FRMPD4, IGF1R, NIPBL, and TMEM63A variants, although the predicted transcript-level consequences differed among them. The complete scores and integrated in silico interpretations are reported in Table 1.

In silico analysis predicted NMD escape for both BCL11B variants and the GPR101 frameshift variant, whereas the remaining truncating variants produced nonconclusive or transcript-dependent predictions (Table 2). These results were not experimentally validated at the RNA level.

### 2.3. Genes Potentially Related to Tooth Agenesis

Among the 28 genes harboring prioritized variants, *FGFR1* and *GLI3* had previously been directly associated with human tooth agenesis or susceptibility to this phenotype. Variants in *BCL11B*, *NIPBL*, and *BBS1* were considered potentially relevant because their associated syndromes may include hypodontia or other dental and craniofacial abnormalities. *PTH1R* and *STAT3* were primarily related to disturbances of tooth eruption rather than to the developmental absence of dental germs. Finally, *BPTF* and *FGFR3* were regarded as preliminary candidates based on their roles in craniofacial development, although a direct association with tooth agenesis has not been established. The remaining genes had no currently recognized relationship with dental agenesis and were interpreted mainly in the context of the patients’ neurological or neurodevelopmental phenotypes.

### 2.4. Descriptive Functional Annotation of Candidate Genes

Descriptive functional annotation of the predefined subset of genes with established or potential relevance to odontogenesis identified shared annotations involving skeletal system development, organ morphogenesis, head development, cartilage and connective-tissue development, chondrocyte differentiation, and endochondral ossification. *FGFR1*, *FGFR3*, *GLI3*, and *PTH1R* were jointly annotated to processes involving cartilage development, chondrocyte differentiation, connective-tissue development, and endochondral ossification, whereas *BBS1*, *BCL11B*, *BPTF*, *NIPBL*, and *STAT3* were predominantly associated with broader developmental processes, including organ morphogenesis, head development, and central nervous system development. These shared annotations provide descriptive biological context for the selected genes but were not interpreted as independent statistical evidence supporting their involvement in tooth agenesis. The annotated biological terms and associated genes are summarized in Table 3.

## 3. Discussion

In this descriptive case series, we performed WES in a clinically heterogeneous cohort of ten individuals with TA and neurodevelopmental or neurodegenerative disorders and descriptively characterized the identified genetic variants. Dental examination identified hypodontia in nine patients and oligodontia, defined by the congenital absence of seven permanent teeth, in one patient. Trio-based or family-based WES identified 30 prioritized variants involving 28 genes. Among these, variants were detected in several genes previously implicated in odontogenesis or in syndromic conditions that may include dental abnormalities, particularly *BCL11B*, *FGFR1*, *GLI3*, *NIPBL*, *BBS1*, *PTH1R*, and *STAT3*. However, segregation patterns, inheritance models, variant classifications, and concordance with the patients’ broader phenotypes differed considerably among these findings. Therefore, rather than identifying a single recurrent molecular cause, our results illustrate the substantial genetic and clinical heterogeneity underlying tooth agenesis in individuals with complex developmental phenotypes. The findings involving *BCL11B* represent one of the most relevant observations in the present cohort. Two unrelated girls with hypodontia and intellectual or neurodevelopmental disability carried distinct heterozygous loss-of-function variants: c.2272del p.Asp758Thrfs54 and c.2132G>A p.Trp711Ter. Pathogenic *BCL11B* variants cause autosomal dominant developmental disorders characterized by variable combinations of intellectual disability, speech impairment, craniofacial dysmorphism, and immunological abnormalities (MIM #617237 and MIM #618092). Dental manifestations—including neonatal teeth, microdontia, enamel defects, hypodontia, and oligodontia—have been reported, but remain incompletely characterized within the *BCL11B*-related phenotypic spectrum [18,19,20]. The identification of two independent truncating variants in patients sharing both neurodevelopmental impairment and hypodontia provides additional clinical evidence supporting dental agenesis as a component of *BCL11B*-related disorders. In silico transcript analysis predicted NMD escape for both BCL11B variants. This finding suggests that the mutant transcripts may remain detectable and potentially encode truncated proteins; however, the prediction does not establish the underlying molecular mechanism, which requires RNA- and protein-level validation. Nevertheless, the small number of observations does not establish a specific genotype–dental phenotype correlation, and systematic dental assessment of larger *BCL11B* cohorts will be required to determine its prevalence and expressivity.

The family comprising patients P7 and P8 provides an informative example of the importance of segregation analysis. P7 carried the novel heterozygous *FGFR1* variant c.553A>C p.Asn185His. *FGFR1* encodes a receptor tyrosine kinase with established roles in craniofacial, skeletal, neural, and dental development. Pathogenic germline variants cause several predominantly autosomal dominant disorders, including Hartsfield syndrome, hypogonadotropic hypogonadism with or without anosmia, and craniosynostosis-related phenotypes. Rare *FGFR1* variants have also been reported in individuals and families with tooth agenesis, supporting its involvement in human odontogenesis [21,22,23]. However, the p.Asn185His variant was absent in P8, the similarly affected son of P7, excluding this variant as the sole explanation for the familial recurrence of hypodontia. A contribution to the phenotype of P7 cannot be excluded but remains unproven. The GPR101 frameshift variant c.1293dup was hemizygous in P7 and heterozygous in P8, who has a confirmed 47,XXY karyotype consistent with Klinefelter syndrome. Although this chromosomal constitution makes paternal transmission biologically possible, the absence of maternal testing and formal parental-origin analysis prevents confirmation of the variant’s origin. Moreover, loss-of-function variants in GPR101 have not been established as a cause of tooth agenesis. The *GPR101* frameshift variant was also predicted to escape NMD; however, its actual effect on transcript stability and protein expression remains unknown. Established *GPR101*-related phenotypes, including X-linked acrogigantism and growth hormone-secreting pituitary adenoma 2 (MIM #300943), are generally associated with increased gene dosage or activating mechanisms rather than loss of function. Although growth hormone excess may cause mandibular overgrowth, interdental spacing, malocclusion, and altered eruption, these manifestations are distinct from congenital tooth absence [24,25,26]. Accordingly, the shared *GPR101* variant should be regarded as an unsolved candidate finding rather than evidence of a novel gene–disease relationship. Its interpretation requires segregation in additional relatives, detailed endocrine evaluation, and functional assessment of the frameshift allele. P8 additionally carried the canonical splice-acceptor variant *NIPBL* c.7861-2A>G. Pathogenic heterozygous variants in *NIPBL* cause Cornelia de Lange syndrome 1 (MIM #122470), in which dental and craniofacial abnormalities may include micrognathia, highly arched palate, small or widely spaced teeth, delayed eruption, and occasionally hypodontia. The position of c.7861-2A>G is compatible with disruption of normal splicing, although RNA studies would be needed to demonstrate its molecular consequence. Because this variant was detected only in P8, it cannot explain the recurrence of hypodontia in both father and son. Nevertheless, it may have contributed to the dental or broader developmental phenotype of P8, particularly if additional clinical manifestations consistent with a mild NIPBL-related disorder are present. This family therefore exemplifies a potentially multilocus architecture in which different variants may contribute to overlapping components of the phenotype without any single finding fully explaining the familial dental presentation.

The homozygous *GLI3* c.2179G>A p.Gly727Arg variant identified in P2 initially appeared biologically relevant because *GLI3* is a central mediator of Sonic Hedgehog signaling and contributes to craniofacial patterning and odontogenesis. Experimental and association studies have implicated *GLI3* in tooth development, root morphogenesis, craniofacial morphology, and susceptibility to tooth agenesis [27,28,29]. However, gene-level biological plausibility must be distinguished from variant-level pathogenicity. The p.Gly727Arg substitution was inherited from two heterozygous parents and is currently classified as benign in ClinVar for postaxial polydactyly, although this classification is based on limited submitted evidence. Furthermore, P2 did not exhibit the characteristic polydactyly or congenital malformation spectrum of an autosomal dominant *GLI3*-related disorder. Consequently, the present observation does not provide sufficient evidence that p.Gly727Arg caused the patient’s hypodontia. It may instead represent a benign allele detected in a biologically plausible candidate gene. This finding illustrates the risk of attributing causality on the basis of gene function alone.

The patient with oligodontia (P4) carried heterozygous variants in *BBS1*, *PTH1R*, and *STAT3*. The recurrent *BBS1* p.Met390Arg variant is a well-established pathogenic allele for autosomal recessive Bardet–Biedl syndrome 1 (MIM #209900), a ciliopathy that may include hypodontia, microdontia, dental crowding, and short tooth roots [30,31]. Nevertheless, no second pathogenic BBS1 allele was identified, making the patient a heterozygous carrier and preventing attribution of the oligodontia to *BBS1*. Similarly, the *PTH1R* p.Thr435Met variant is of uncertain significance, and the established *PTH1R*-related dental phenotype is primary failure of tooth eruption rather than tooth agenesis [32,33]. *STAT3*-related hyper-IgE syndrome is also more commonly associated with retention of deciduous teeth and delayed eruption than with congenital tooth absence [34,35]. Moreover, the patient’s diagnosis of Down syndrome represents an important alternative contributor to his dental phenotype. Therefore, none of these three variants independently provides a convincing explanation for the oligodontia, and an oligogenic or modifying effect remains speculative. The descriptive functional annotation indicated that several candidate genes participate in overlapping developmental processes involving skeletal, craniofacial, cartilage, and connective-tissue development. This functional overlap may provide biological context for discussing the identified genes but does not validate their selection or establish their involvement in tooth agenesis. Indeed, because the gene set was preselected partly on the basis of its potential relevance to odontogenesis, the observed developmental annotations are inherently influenced by the selection strategy and should not be regarded as independent evidence of pathogenicity or causality.

Several aspects contribute to the descriptive value of the present study. First, the combination of specialist dental assessment with genomic investigation enabled congenital tooth absence to be distinguished from eruption abnormalities, an essential distinction for interpreting genes such as *PTH1R* and *STAT3*. Second, trio- and family-based analysis allowed evaluation of inheritance and cosegregation. In particular, the absence of the *FGFR1* variant in P8 prevented an otherwise plausible but unsupported causal interpretation. Third, the identification of two distinctive loss-of-function *BCL11B* variants in unrelated patients with hypodontia and neurodevelopmental abnormalities is consistent with the dental manifestations previously reported in *BCL11B*-related disorders. However, this observation requires replication and does not establish a causal relationship with hypodontia. Descriptive functional annotation further identified developmental processes shared by several selected genes, although these findings were not regarded as independent evidence of their involvement in TA. The broad WES approach also provided information beyond the primary dental phenotype. In patients with complex neurodevelopmental presentations, variants not directly related to tooth agenesis may help explain other components of the phenotype or identify findings requiring clinical reassessment. When appropriately validated and interpreted within the patient’s phenotype, pathogenic or likely pathogenic additional findings may inform genetic counselling, specialist referral, surveillance, or preventive care. Variants of uncertain significance should not independently guide medical management, but their identification may support phenotype-directed follow-up, segregation analysis, and periodic reinterpretation as new evidence becomes available. Thus, the clinical value of WES in this cohort extends beyond establishing a single diagnosis and lies partly in enabling multidisciplinary and longitudinal genomic evaluation.

The study nevertheless has important limitations. The cohort was small, recruited at a single institution, and clinically selected for the coexistence of tooth agenesis and neurodevelopmental phenotypes, increasing the possibility of ascertainment bias and coincidental findings. Moreover, the study was not sufficiently powered to estimate variant frequencies, diagnostic yield, or statistical associations with the dental phenotype. Functional validation was not available for the novel missense, frameshift, or splice-site variants, thereby precluding conclusions regarding their biological effects or causal contribution to tooth agenesis. Although the dental phenotype was confirmed through clinical examination, radiographic assessment, and dental history, the wide age range of the participants may have affected the evaluation of additional age-dependent dental manifestations. WES also has limited sensitivity for structural, noncoding, repeat-associated, and some copy-number variants. Finally, because the functional annotation was performed on a preselected set of genes with established or potential relevance to odontogenesis, the resulting biological terms should be considered descriptive and cannot be interpreted as independent evidence supporting their involvement in tooth agenesis.

Overall, this study should be interpreted as a descriptive WES case series of clinically selected patients with tooth agenesis and associated neurodevelopmental phenotypes. The identified variants represent candidate or clinically relevant findings and do not, by themselves, demonstrate a causal contribution to tooth agenesis. Among them, the identification of two distinct pathogenic or likely pathogenic loss-of-function variants in *BCL11B* in two unrelated patients with hypodontia represents the most compelling observation, given the dental abnormalities previously reported in *BCL11B*-related disorders. Nevertheless, the limited cohort size, its clinically selected composition, and the absence of functional validation prevent definitive gene–phenotype conclusions. The remaining variants should therefore be interpreted cautiously and require confirmation through segregation studies, functional investigations, and replication in larger independent cohorts. Within these limitations, this case series illustrates the value of WES for the comprehensive characterization of genetically and clinically heterogeneous patients and for generating hypotheses concerning the molecular basis of tooth agenesis.

## 4. Materials and Methods

### 4.1. Patients Enrollment

Patients were recruited at the IRCCS Oasi Research Institute of Troina, Italy, a specialized research and healthcare center for rare genetic disorders associated with neurodevelopmental and neurodegenerative conditions. Recruitment was conducted between January 2024 and July 2026 following referral by the dentist of the Institute, who identified congenital dental abnormalities during routine clinical evaluation. Eligibility criteria included a clinical diagnosis of tooth agenesis, categorized as hypodontia, oligodontia, or anodontia. Hypodontia was defined as the congenital absence of one to five permanent teeth, excluding third molars; oligodontia as the absence of six or more permanent teeth, excluding third molars; and anodontia as the complete absence of teeth. Dental agenesis was assessed by clinical dental examination. All enrolled individuals had an underlying neurodevelopmental or neurodegenerative condition and had previously been referred for genetic investigation. Therefore, the study population represents a clinically selected cohort and should not be considered representative of individuals with isolated tooth agenesis in the general population. Demographic, neurological, dysmorphological, and dental findings were collected from the patients’ clinical records and encoded, where applicable, using Human Phenotype Ontology terms.

This study was conducted in accordance with the Declaration of Helsinki and was approved by the Ethics Committee of the IRCCS Oasi Research Institute of Troina (approval code: 2022/04/05/CE-IRCCS-OASI/52). Written informed consent for genetic testing and the use of anonymized clinical and genetic data for research and publication was obtained from the participants or their legal guardians.

### 4.2. Assessment of Dental Agenesis

Tooth agenesis was assessed by an experienced dentist through clinical examination, review of the individual dental history, and evaluation of radiographic documentation. A permanent tooth was considered congenitally absent when the corresponding tooth germ was not radiographically detectable and there was no history of extraction or other acquired tooth loss. Radiographic examination was also used to exclude delayed eruption and impaction. Missing permanent teeth were recorded according to the two-digit Fédération Dentaire Internationale (FDI) notation.

### 4.3. WES and Variant Analysis

Genomic DNA was extracted from peripheral blood leukocytes obtained from each proband and available family members using a standardized protocol [36]. WES was performed in all affected participants and available parents. Trio-based WES was performed for P2, P3, and P5. Duo analysis was performed for P1 and his available mother and for P8 and his affected father, P7, who was also independently included in the cohort. Proband-only WES was performed for P4, P6, P7, P9, and P10 because parental samples were unavailable. No relatives underwent targeted segregation testing alone. Patient-level inheritance data are reported in Supplementary Table S2. Exome sequencing was performed using libraries prepared with the Agilent SureSelect Human All Exon V7 enrichment kit (Agilent Technologies, Santa Clara, CA, USA), according to the manufacturer’s instructions. Sequencing was carried out on an Illumina HiSeq 3000 platform (Illumina, San Diego, CA, USA), with at least 20× coverage achieved for 97% of the targeted regions. Raw sequence reads were aligned to the GRCh38/hg38 human reference genome using BWA-MEM, and single-nucleotide variants and small insertions/deletions were called using GATK HaplotypeCaller according to the GATK Best Practices workflow. Only variants passing the quality-control filters implemented in the GATK Best Practices workflow were retained for downstream analysis. Sequence variant nomenclature was reported according to Human Genome Variation Society recommendations. Variants were initially filtered using a minor allele frequency threshold below 1% in population databases, including the 1000 Genomes Project, ESP6500, ExAC, and gnomAD. This threshold was applied as an initial screening criterion to account for population-specific genetic variability. Variants that were absent or extremely rare in these databases were subsequently prioritized. Variant prioritization considered the presumed inheritance model—including de novo, autosomal recessive, and X-linked inheritance—the predicted functional consequence, gene–disease associations, and consistency with the patient’s clinical phenotype. Transcript annotation was based preferentially on the MANE Select transcript. When no MANE Select transcript was available, the transcript associated with the established gene–disease relationship or the principal clinically relevant transcript was used. Phenotype-driven analysis was performed using the Human Phenotype Ontology terms provided by the referring clinicians. Particular attention was given to genes previously associated with tooth development, tooth agenesis, craniofacial abnormalities, or syndromic conditions involving dental anomalies. Variant prioritization was performed using Franklin by QIAGEN (Hilden, Germany). Candidate variants were visually inspected using the Integrative Genomics Viewer and subsequently confirmed by Sanger sequencing.

### 4.4. Data Analysis

Sequence variants were annotated and prioritized using the Franklin platform (Genoox, Tel-Aviv, Israel). Prioritization was based on population allele frequency, predicted molecular consequence, inheritance pattern, available clinical and functional evidence, and consistency with the patient’s phenotype. Variants were classified as pathogenic, likely pathogenic, of uncertain significance, likely benign, or benign according to the American College of Medical Genetics and Genomics and the Association for Molecular Pathology (ACMG/AMP) guidelines [37]. The criteria suggested by the platform were subsequently reviewed in the context of the available clinical, segregation, and literature data. The potential effects of splice-region variants were evaluated using SpliceAI and Pangolin through the SpliceAI Lookup web server (https://spliceailookup.broadinstitute.org/; accessed on 12 September 2026). Predicted transcript-level consequences were further assessed using the SAI-10k-calc output provided by the same platform. These analyses were considered supportive computational evidence and not a substitute for RNA-based functional validation. Potential splice-altering variants were evaluated using SpliceAI and Pangolin through the SpliceAI Lookup web server (https://spliceailookup.broadinstitute.org/) (accessed on 12 September 2026). Nonsense-mediated mRNA decay (NMD) was assessed for truncating variants using NMDEscPredictor (https://nmdprediction.shinyapps.io/nmdescpredictor/) (accessed on 12 September 2026) and the Ensembl Variant Effect Predictor NMD plugin (https://www.ensembl.org/tools/vep/) (accessed on 12 September 2026). Predictions were interpreted according to the clinically relevant transcript and considered conclusive only when the transcript and HGVS annotation matched those reported in the study.

### 4.5. Functional Annotation of Candidate Genes

The genes selected for their potential relevance to odontogenesis were functionally annotated using g:Profiler with *Homo sapiens* as the reference organism, considering Gene Ontology Biological Process, Reactome and WikiPathways annotations. Because this gene set had been preselected partly on the basis of its known or proposed involvement in dental and craniofacial development, the analysis was used exclusively to summarize shared biological annotations. No inferential conclusions regarding pathway enrichment, variant pathogenicity, or gene–disease causality were drawn from this analysis.

## 5. Conclusions

This descriptive WES case series illustrates the clinical and genetic heterogeneity of tooth agenesis in patients with concomitant neurodevelopmental disorders. Although several prioritized variants were identified in genes involved in craniofacial, skeletal, and dental development, the available genetic and clinical evidence does not establish these variants as causal contributors to tooth agenesis. The identification of two distinct pathogenic or likely pathogenic loss-of-function variants in *BCL11B* in two unrelated patients with hypodontia represents the most compelling observation of this study. This finding is consistent with the dental abnormalities previously reported in *BCL11B*-related disorders and supports further investigation of the dental phenotype associated with this gene. Nevertheless, the limited cohort size and the absence of functional validation preclude definitive conclusions regarding its contribution to tooth agenesis. The remaining findings, including variants in *FGFR1*, *GPR101*, *NIPBL*, *GLI3*, *BBS1*, *PTH1R*, and *STAT3*, should be regarded as candidate or clinically relevant observations requiring confirmation through additional segregation studies, functional analyses, and larger independent cohorts. Overall, the study highlights the value of WES for the comprehensive genetic characterization of clinically complex patients while emphasizing the need to distinguish the general clinical significance of a variant from its specific relevance to dental development.

## Figures and Tables

**Table 1:** In silico characterization of splice-region variants identified by whole-exome sequencing. SpliceAI and Pangolin were used to predict alterations of canonical splice sites and the potential activation of cryptic sites. The predicted transcript-level consequences were assessed using SAI-10k-calc. Scores, predicted splice alterations, and their integrated in silico interpretation are reported for each variant. These computational predictions were not confirmed by RNA analysis.

Gene	Variant	SpliceAI Prediction	Pangolin Prediction	Integrated In Silico Interpretation
FRMPD4	c.3964+2T>C	Donor loss: 0.97 (−2 bp); donor gain: 0.27 (+100 bp)	Splice loss: 0.70 (−2 bp); splice gain: 0.22 (+100 bp)	Strong concordant evidence of canonical donor-site disruption, with possible activation of a cryptic donor site. SAI-10k-calc predicted retention of 102 coding nucleotides from intron 16, resulting in an in-frame transcript alteration; NMD is therefore not expected for this predicted event.
IGF1R	c.3722+5G>A	Donor loss: 0.60 (−5 bp); donor gain: 0.06 (+129 bp); acceptor loss: 0.16 (+338 bp)	Splice loss: 0.74 (−5 bp); splice gain: 0.26 (+129 bp)	Concordant evidence of donor-site disruption, potentially accompanied by activation of a cryptic donor site. However, the resulting aberrant transcript could not be reliably defined.
NIPBL	c.7861-2A>G	Acceptor loss: 1.00 (2 bp); acceptor gain: 0.70 (+39 bp)	Splice loss: 0.91 (2 bp); splice gain: 0.63 (+39 bp)	Very strong concordant evidence of canonical acceptor-site disruption and cryptic acceptor activation. SAI-10k-calc predicted partial deletion of exon 46 involving either 37 or 40 coding nucleotides, both resulting in a frameshift.
TMEM63A	c.1571+3A>G	Donor loss: 0.89 (3 bp); acceptor loss: 0.79 (+89 bp); donor gain: 0.01; acceptor gain: 0.01	Splice loss: 0.73 (3 bp); splice gain: 0.04 (1 bp)	Concordant evidence of donor-site disruption. SAI-10k-calc predicted exon 17 skipping, removing 87 coding nucleotides and producing an in-frame transcript alteration; NMD is therefore not expected for this predicted event.

SpliceAI and Pangolin predictions were generated using the indicated RefSeq transcripts. Predicted transcript consequences were evaluated using SAI-10k-calc. All reported effects are computational predictions and were not confirmed by RNA analysis. Positions are reported relative to the affected or predicted splice site, as provided by the annotation platform.

**Table 2:** In silico NMD predictions for truncating variants identified by WES.

Gene	Variant	Protein Change	In Silico NMD Prediction
*BCL11B*	c.2272del	p.(Asp758ThrfsTer54)	Predicted NMD escape
*BCL11B*	c.2132G>A	p.(Trp711Ter)	Predicted NMD escape
*GPR101*	c.1293dup	p.(Pro432ThrfsTer31)	Predicted NMD escape
*GJB2*	c.35del	p.(Gly12ValfsTer2)	No confident prediction; single coding exon
*TGM6*	c.154G>T	p.(Glu52Ter)	Not annotated as NMD escaping
*LPA*	c.1173C>A	p.(Cys391Ter)	Not annotated as NMD escaping
*TRPM3*	c.4012dup	p.(Ser1338PhefsTer33)	Inconclusive; transcript-dependent annotation
*PKP2*	c.1881del	p.(Lys628ArgfsTer12)	Inconclusive; transcript mismatch

NMD predictions were obtained using NMDEscPredictor and the Ensembl VEP NMD plugin. Results are computational predictions without RNA-level validation. Absence of an NMD-escape annotation does not constitute experimental evidence of transcript degradation.

**Table 3:** Descriptive functional annotation of candidate genes with established or potential relevance to odontogenesis. Selected Gene Ontology Biological Process and WikiPathways annotations are reported together with the associated candidate genes. These annotations are presented solely to provide biological context. Because the genes were preselected partly according to their known or proposed relevance to dental and craniofacial development, the reported associations should not be interpreted as independent statistical evidence of enrichment, pathogenicity, or causality.

Term ID	Annotated Biological Process/Pathway	Associated Candidate Genes
GO:0001501	Skeletal system development	*BBS1*, *FGFR1*, *FGFR3*, *GLI3*, *NIPBL*, *PTH1R*
GO:0009887	Animal organ morphogenesis	*BBS1*, *BCL11B*, *FGFR1*, *FGFR3*, *GLI3*, *NIPBL*, *STAT3*
GO:0007417	Central nervous system development	*BBS1*, *BCL11B*, *BPTF*, *FGFR1*, *GLI3*, *NIPBL*, *STAT3*
GO:0051216	Cartilage development	*BBS1*, *FGFR1*, *FGFR3*, *GLI3*, *PTH1R*
GO:0007423	Sensory organ development	*BBS1*, *BCL11B*, *FGFR1*, *GLI3*, *NIPBL*, *STAT3*
GO:0061448	Connective tissue development	*BBS1*, *FGFR1*, *FGFR3*, *GLI3*, *PTH1R*
GO:0002062	Chondrocyte differentiation	*FGFR1*, *FGFR3*, *GLI3*, *PTH1R*
WP:WP4808	Endochondral ossification with skeletal dysplasias	*FGFR1*, *FGFR3*, *GLI3*, *PTH1R*
WP:WP474	Endochondral ossification	*FGFR1*, *FGFR3*, *GLI3*, *PTH1R*
GO:0060322	Head development	*BBS1*, *BCL11B*, *BPTF*, *FGFR1*, *GLI3*, *NIPBL*

## Data Availability

The original contributions presented in this study are included in the article. Further inquiries can be directed to the corresponding author.

## Supplementary Materials

The following supporting information can be downloaded at https://www.mdpi.com/article/10.3390/ijms27209357/s1.

## Abbreviations

The following abbreviations are used in this manuscript:

ASD	Autism spectrum disorder
DS	Down syndrome
ID	Intellectual disability
NDD	Neurodevelopmental disorder
NGS	Next generation sequencing
TA	Tooth agenesis
VUS	Variant of uncertain significance
WES	Whole exome sequencing
WGS	Whole genome sequencing
